# Understanding gambling related harm: a proposed definition, conceptual framework, and taxonomy of harms

**DOI:** 10.1186/s12889-016-2747-0

**Published:** 2016-01-27

**Authors:** Erika Langham, Hannah Thorne, Matthew Browne, Phillip Donaldson, Judy Rose, Matthew Rockloff

**Affiliations:** 1 School of Human, Health and Social Sciences, CQ University, PO Box 7815, Cairns, QLD 4870 Australia; 2 School of Human, Health and Social Sciences, CQ University, 120 Spencer Street, Melbourne, VIC 3000 Australia; 3 School of Human, Health and Social Sciences, CQ University, Locked Bag 333, Bundaberg, QLD 4670 Australia

**Keywords:** Gambling, Gambling harm, Problem gambling, Taxonomy, Public health, Summary measure, Conceptual framework

## Abstract

**Background:**

Harm from gambling is known to impact individuals, families, and communities; and these harms are not restricted to people with a gambling disorder. Currently, there is no robust and inclusive internationally agreed upon definition of gambling harm. In addition, the current landscape of gambling policy and research uses inadequate proxy measures of harm, such as problem gambling symptomology, that contribute to a limited understanding of gambling harms. These issues impede efforts to address gambling from a public health perspective.

**Methods:**

Data regarding harms from gambling was gathered using four separate methodologies, a literature review, focus groups and interviews with professionals involved in the support and treatment of gambling problems, interviews with people who gamble and their affected others, and an analysis of public forum posts for people experiencing problems with gambling and their affected others. The experience of harm related to gambling was examined to generate a conceptual framework. The catalogue of harms experienced were organised as a taxonomy.

**Results:**

The current paper proposes a definition and conceptual framework of gambling related harm that captures the full breadth of harms that gambling can contribute to; as well as a taxonomy of harms to facilitate the development of more appropriate measures of harm.

**Conclusions:**

Our aim is to create a dialogue that will lead to a more coherent interpretation of gambling harm across treatment providers, policy makers and researchers.

## Background

The existence of gambling related harm is well established. There are common negative impacts associated with participation in gambling, and greater and more severe harms when gambling frequently and with more money. Public health approaches to gambling in terms of prevention and treatment of problems with gambling make reference to harm minimisation. However, this term is somewhat ambiguous due to the lack of: (a) a consistent definition of gambling related harm, (b) conceptualisation of the breadth and experience of harm, and (c) an appropriate means of measuring harm.

Whilst there is no single internationally agreed-upon definition of harm in relation to gambling, there are consistent patterns of interpretation throughout the literature that suggest some degree of convergence in the understanding of gambling-harm. Unlike indicators of gambling disorders or problematic behaviours, measures that specifically target gambling harm are under-developed. To a large degree, this reflects an emphasis on diagnosis or screening for problem gambling; rather than on measuring the range of negative outcomes that can arise from gambling behaviours, whether symptomatic of addiction or not.

Harms from gambling are varied and diffuse, unlike the more direct and tractable harms caused by physical illnesses or even substance abuse. Additionally, the large number of potential harms that may not be easily and unambiguously traced to gambling as their source, impacts on efforts to address gambling harm from a public health perspective. The current measurements used are inappropriate and insufficient, being most typically proxies of harm that come from gambling behaviour prevalence measures or unsystematic explorations of harms within the context of specific research studies. These approaches lack content validity, construct validity or both.

### Definitions of gambling related harm

Harm is a term that is immediately intuitive, implying damage and adverse consequences. However, the assumption that it is unnecessary to define the term precisely in relation to gambling is mistaken. Neal et al [1], in developing a national definition for problem gambling and harm, acknowledged the issue of lacking a clear definition of gambling-related harm. This lack of a robust, agreed upon definition may reflect the multi-disciplinary interest in the phenomena of gambling, and the differences in approach and perspective on gambling from these different disciplines [1]. Arguably, the notion that harms arise from uncontrolled, addictive or problematic gambling behaviour has historically been treated as implicit, based on either self-assessment, help seeking behaviours, or clinical diagnosis that suggest harmful consequences have occurred. However the absence of a detailed and explicit definition, with an accompanying conceptual model, makes it difficult to operationalize the concept and thereby measure the impacts or severity of harm experienced [1], and this deficit separates gambling from other public health issues to its detriment.

Neal et al [1] identified two definitions of gambling harm: one from the Queensland Government [2], and one from the New Zealand Gambling Act (2003) [3]. The Queensland definition describes harm as a ‘range of adverse consequences’, in which ‘the safety or wellbeing of gambling consumers or their family or friends are placed at risk’ and/or negative impacts extending to the broader community. In describing harm as a set of impacts and consequences, the Queensland definition is clear that gambling harms are the outcome of problematic gambling, rather than problematic gambling itself. However, they limit harm to occurring only from problematic gambling and in describing safety and risk in relation to the product, the Queensland definition would appear to be focused on a product-safety paradigm of evaluating the hazard involved in consumption of commercial gambling which is inconsistent with a social model of health. The New Zealand 2003 Gambling Act definition is broader, describing harm as ‘any kind of harm or distress arising from, or caused or exacerbated by, a person’s gambling’. This definition includes psychological or emotional impacts of gambling, as well as presumably more concrete forms of harm, such as financial loss. This is emphasised in the second part of the definition, which explicitly refers to personal, social or economic harms. The New Zealand definition also emphasises the multiple social scales at which harm can take place, which is more consistent with a social model of health, enumerating four levels at which harm may occur: the individual person, spouse, family, whanau, or wider community, in the workplace, or in society at large.

Neal et al [1] were critical of both definitions for being too vague to be useful for operationalizing the concept of gambling harm for the purpose of measurement. Similar limitations were later noted by Currie et al [4]. The Queensland Government definition does not make any reference as to the mechanism by which harms occur. However, the New Zealand definition does offer an important insight in terms of suggesting that gambling can exacerbate, as well as generate harms. This is an important point, as gambling harms rarely occur in isolation. Rather, one of the key features of gambling problems is co-morbidity with a range of other harmful behaviours or reduced health states, such as alcohol use and depression [5, 6]. Importantly, both definitions describe harm as extending beyond the individual to the family, friends and community.

In the literature since Neal et al [1] and Currie et al [4], harm still has not been defined, but harmful behaviour is either explicitly or implicitly referred to as having negative consequences and thus these negative consequences are the harm caused by the behaviour (gambling). To add further uncertainty, the term harm is often used interchangeably to refer to the behaviour - not just the consequence - and is used in multiple items on screening instruments such as the PGSI [7]. However, conflation of the harm (outcome) with the source (problematic behaviour) is not isolated to gambling, and is consistent with other public health literature, for example, alcohol [8].

### Measurement of harm

The limitations and relative lack of progress in defining or conceptualising harm is reflected in how harm is currently measured in the literature. This separates gambling from other public health issues, which utilise summary measures to quantify the impact on population health. Currie et al [4] identified three sources that the measurement of harms have been derived from: 1) diagnostic criteria of pathological or problem gambling, 2) behavioural symptoms associated with disordered gambling, and 3) the negative consequences experienced. All three of these sources might be criticised for failing to capture the breadth and complexity of harm to the person who gambles, or the experience of harm beyond the person who gambles.

Firstly, the usefulness of diagnostic criteria to measure harm is limited. It restricts the focus to people experiencing problems with gambling, failing to recognise that harm occurs across the spectrum of gambling behaviour and severity. This is common in treatment, policy and empirical research, which led the Productivity Commission [9] to raise concerns that the smaller, but more prevalent harms that are being ignored can aggregate to a significant population level harm.

The second category of measures in the literature is the use of behavioural symptoms to measure harm. Symptomatology does have a strong relationship with harm, and behavioural indicators are of importance in their own right in clarifying the mechanisms by which harm arises. However, as when using diagnostic criteria, a symptoms-based measure of harm (e.g., lying to someone about gambling) is more precisely a behavioural proxy measure, and does not necessarily provide a stable and precise measure of gambling harm.

The third category, the experience of negative consequences, is the closest approximation of harm due to its focus on outcomes [4]. Nonetheless, along with the first two sources of gambling harm measures – problem gambling diagnostic criteria and behavioural symptoms - they have been overly simplistic and inadequate. There are a number of limitations to these types of measures that reduces their utility, including the lack of scale of the impact of that harm or a consistency of measures across surveys that would allow the comparison of impact across populations or time. For example, gambling expenditure is a common negative consequence used as a proxy indicator for harm [10] and whilst a strong relationship between expenditure and harm has been demonstrated [4] these measures are normally based on aggregated data that cannot provide detail on comparison to discretionary income, impact, or vulnerability and the individual level necessary to demonstrate causality.

### Efforts to conceptualise harm in relation to gambling

Abbot et al’s [11] Conceptual Framework for Factors Influencing Harmful Gambling made an important distinction between gambling behaviour and gambling related harm. An important difference in this framework is the division of gambling into harmful and non-harmful, rather than problem and recreational, and the authors make the point that the difference between these is related to severity and frequency [11]. The framework also separates harmful gambling from problem gambling status and broadens the focus to consequences beyond the person who gambles, to include family, social networks and community. Consistent with both a public health approach and a social model of health, Abbott et al.’s [11] framework recognises the complexity of factors that drive the phenomenon rather than focussing on simplified causal pathways. The framework provides a conceptual model of understanding the inputs or environmental context to harmful gambling, but does not address the manifestation of those harms. It is this existing gap in our understanding of the manifestation or experience of harms that the present study seeks to address.

The purpose of this paper is threefold. Firstly, it proposes a functional definition of gambling related harm that can be operationalised to support the measurement of gambling related harm consistent with standard epidemiological protocols used in public health. Secondly, it contributes a conceptual framework for gambling related harm as a consequence or outcome that captures the breadth of how harms can manifest for the person who gambles, their affected others and their communities consistent with social models of health. Finally it identifies a taxonomy of harms utilising the conceptual framework experienced by the person who gambles, affected others, and the broader community. Both the conceptual framework and proposed definition are aimed at an intended audience of researchers, treatment providers and those involved in developing public policy related to gambling, whilst remaining consistent with the national definition of problem gambling. The proposed framework and taxonomy are based on the literature on gambling harms and consultation with experts and community sources described in the next section.

## Methods

Data regarding harms from gambling was gathered using four separate methodologies. Initial data was gathered from a literature review to examine the types of harm experienced from gambling. Focus groups and interviews (*n* = 35) were then conducted with professionals involved in the provision of problem gambling treatment, ancillary counselling services (finance, relationship or mediation), community education, primary health care, public policy, research and the provision or promotion of responsible gambling within venues. Participants were systematically recruited via email contact with organisations within Victoria that provided gambling treatment, financial counselling or emergency welfare support. A snowball technique was also used to leverage off informal networks and identify potential participants that may not have been known to the researchers or not currently employed within the identified organisations. The focus groups were conducted in person, and the interviews were conducted both in person and via telephone. Focus groups averaged around 90 min in length and interviews around 40 min. This phase was followed by semi-structured interviews (*n* = 25) with individuals who identified that they had experienced harm from either their own and/or someone else’s gambling. Individuals were recruited using advertising on social media, and all interviews were conducted via telephone. Participants identified as either people who gambled (*n* = 11), affected others (*n* = 9) or both a person who gambles and affected other (*n* = 5). These interviews ranged from twenty to sixty minutes in length and participants were compensated for their time with a store voucher.

A limitation of interviews is the potential for participants not to disclose sensitive or stigmatized information when being personally identified due to social desirability bias. Accordingly, public internet gambling help or support forums (*n* = 469 forum posts) were examined to identify any further themes or harms that had not been captured during the literature review or the consultative phases and validate the proposed taxonomy of harms. This form of unobtrusive method was utilised to source existing records of people’s lived experience of harm. Ethical clearance for all of these stages was gained from CQ University Human Research Ethics Committee, clearance reference H14/06-142. All participants provided informed consent prior to data collection.

Focus group and interview data was transcribed verbatim, checked for accuracy and anonymised then uploaded into NVivo Software to facilitate coding and analysis. Forum posts from Gambling Help Online forums dating back over five years were accessed during October, 2014 and again in June 2015. Relevant data was imported using NCapture into Nvivo software. Data from each of these stages were analysed sequentially first, and then synthesized across stages. Initial codes developed sequentially from the focus groups, interviews and analysis of forum posts. A grounded theory methodology was utilised; this approach has the capacity to identify how participants have experienced a phenomenon of harm through a process of substantive and theoretical coding and constant comparison of data and concept [12]. Data was coded initially using open coding, utilising in vivo coding to identify how people perceived harm, their experiences of harm, and conceptualisations of harm. Axial coding was then utilised to understand the relationships between the experiences of harm in terms of the domains in which harm occurred and the temporal sequence in which they occurred. These codes underpinned the development of the conceptual framework [13]. Finally, the catalogue of harms identified in the data were organised into a taxonomic structure.

## Results and discussion

### Functional definition of gambling related harm

The concept of harm, whilst intuitive, is also highly subjective, which is reflective of a social model of health. Given this subjectivity, and the differences between disciplines interested in the phenomena of gambling, it is unsurprising that an agreed definition of gambling related harm is yet to be realised. The data gathered for this project highlighted the breadth of experiences of harm across multiple domains of people’s lives, the subjectivity of what people considered harmful to themselves or others, and the complex inter-relationships between harms and sources of harm. Further complexity was identified due to the difficulty in isolating the harm caused specifically by gambling from the influence or interaction of other comorbidities, such as alcohol abuse or depression. However, capturing this subjectivity and complexity was determined not to be the role of a functional definition. The critical function for the definition was its ability to be operationalised in a way that gambling related harm could be measured consistent with other public health issues.

The functional definition of gambling related harm generated from an examination of the data is:

Any initial or exacerbated adverse consequence due to an engagement with gambling that leads to a decrement to the health or wellbeing of an individual, family unit, community or population.

There were a number of factors that drove the wording of the definition that are worth highlighting. Firstly, the definition clearly delineates harm as an outcome, allowing the focus to be on consequences rather than causes or symptoms of harmful gambling. It is explicit in separating this from related, but different, issues such as categorisations of behaviour of gambling, clinical diagnosis, risk factors or the environment in which gambling occurs. Secondly, the definition captures that harm can occur to any person, at any time. It allows for the inclusion of any instance of harm, from the first experience with gambling through to legacy and intergenerational harms, rather than being focussed only on harms experienced from gambling at a diagnostic point of problem gambling or only whilst engaging with gambling. This is an important broadening of focus that assists in addressing gambling related harm from a public health perspective. Thirdly, the definition allows for harm being both subjective and socially constructed, consistent with the World Health Organisation (WHO) definition of health. Fourthly, the definition allows for harms that may occur from engagement with gambling, without having to participate in gambling. This allows for the inclusion of harm to people who work in the gambling industry or are nvolved in treatment and support services accessed by people experiencing problems with gambling. This separates them from the more traditional definition of an affected other and broadens our conceptualisation of gambling related harm from current pathogenic approaches. Finally, the definition is grounded in a public health approach to allow for the operationalisation and future measurement that is consistent with standard public health approaches to measuring health outcomes. It also allows for the influence of comorbidities to be included in those measurements. The use of the word ‘decrement’ captures both the generation and exacerbation of harm related to health and wellbeing, and is consistent with health state valuation calculation methodologies.

### Conceptual framework of gambling related harm

A conceptual framework links discrete concepts based on multiple theories and is seen as an impetus in the development of theory [14]. The proposed conceptual framework of gambling related harm emerged from an inductive analysis and linked several existing theories with generated from the data. Sensitizing concepts from the researchers’ a priori knowledge of the topic provided a starting point [12, 15–17] to understand the experiences of harms (types and breadth) at the three levels of the person who gambles, affected others and broader community. These levels reflect that the person who gambles would most likely be both the first to experience harm (the index case) and would also be expected to experience greater levels of harm. It is not intended to imply that the cause of the harm is the person who gambles. The causal mechanisms are a complex interaction of broad social and environmental determinants. A further sensitizing concept was the notion that smaller harms could occur from any level of engagement or behavioural level of gambling.

Through constant comparison of data and concepts, initial themes of the experience of harms were identified. Two separate groups of themes clearly emerged and the conceptual framework illustrates the relationship between them. The first was that harms could be grouped into clear dimensions or classifications relating to the experience of harm. The second was that of temporal categories in the experience of harm, i.e., harm could occur from the first engagement with gambling and extend beyond engagement with gambling. Moreover, there was often a temporal point of significance in terms of the experience of harm that could be labelled as a crisis.

The classifications represent the different dimensions or domains in which harm occurs whilst the categories captured the temporal experience in which harm occurs. This addressed two of the principle deficiencies identified in the existing conceptualisation of gambling related harm. The framework also assists with the classification and categorisation of experiences of harm for the creation of the taxonomy. Consistent with the guidelines for creating a taxonomy, the division of entities into classifications were mutually exclusive, yet they can cross categorical boundaries. That is, a harm that occurs in the general harm temporal category could also occur during a crisis or as a legacy harm. The framework does not attempt to capture causal sequences or pathways of harms, this would only be possible using a prospective longitudinal methodology.

The data around the temporal experience of harms identified three clear differentiations. The first group to be identified were harms that occurred at a temporal point of significance, often labelled as a crisis. These harms were significant enough to motivate people towards seeking assistance or treatment or attempting to change their behaviour. This was not unexpected given the initial data was gathered from professionals involved in treatment and support services. Similarly the second group to be identified, which were labelled as legacy harms, was also strongly identified in this phase. Legacy harms related to those harms that continue to occur (or emerge) even if the person’s engagement with gambling ceases through changes in their own or someone else’s behaviour, but may also be experienced if a person continues to gamble. The label was chosen to capture the ongoing impact of harm, and to highlight that harm does not cease with the behaviour. Less significant in the early data was detail around the general harms that might occur from someone having an initial engagement with gambling, through to someone who had reached a temporal point of significance. Participants were encouraged to expand on their experiences or recollection of these types of harms given the broad scope of them and previous identification of this gap in the understanding [9].

It is important to highlight that these are temporal categories or differentiations, and do not represent a continuum. This is because gambling is a behaviour, not a disease that follows a particular course. The framework is focussed on consequences of the behaviour and these are separate to the symptoms of the behaviour and diagnostic criteria. The behaviour may be undertaken at different times, and may vary in its intensity on these occassions in a bilateral movement [18, 19]. Regardless of the behaviour or diagnosis at any particular time, the three categories of harm experienced remain valid. For example, a person may have abstained from gambling for some years but still be experiencing legacy harms due to previous engagement with gambling. This is further highlighted in the data with the identification of binge gamblers, people who may not gamble for considerable amounts of time, but will have a night or weekend of gambling at a level that causes harm.

Further analysis of the data identified a final theme relating to lifecourse and intergenerational harms. The position of this on the conceptual framework represents its unique position as both a classification and category. As a classification it represents a unique set of harms that reflects a cumulative yet separate impact to a person who gambles, an affected other, or the broader community. As a category it represents a unique position in terms of time frames, in that it can impact across all three temporal categories, and that intergenerational harm is a pervasive legacy harm that impacts beyond the current lifecourse.

### Classifications of harms

**Fig. 1:**
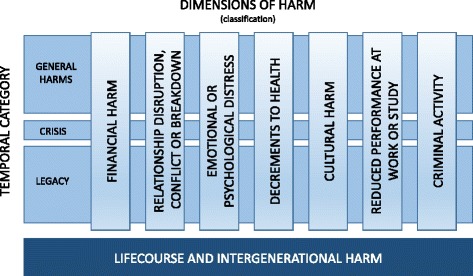
Conceptual Framework of Gambling Related Harm (insert here)

The classifications of harms represents the first theory that was generated from the data, that harm occurs across a broad number of domains within the life of the person who gambles, their family and friends, and the broader community. Initially six different thematic classifications of harm were identified that could occur either sequentially or in parallel: financial harms, those harms relating to relationships, emotional or psychological harms, impacts on the person’s health, impacts on work, study or economic activity, and criminal acts. Further analysis of the data relating to people with strong religious beliefs, CALD groups and indigenous populations identified a seventh classification of harm: cultural harms. These emerged as separate to the relationship harms, although they tend to occur together due to the strong link to culture through family and other relationships. The conceptual framework is illustrated at Fig. 1, and the classifications are discussed in detail below.

The classifications of harm possess the five attributes required for generating a classification for a taxonomy [20]. Firstly, the classifications must be mutually exclusive, that is it must not be possible for an entry into one classification to be included in another. Secondly, the items in each classification should be homogenous, being more similar to each other than to items in other classifications. Thirdly, they should be exhaustive, although some writers argue it is not possible for our knowledge to be totally exhaustive [21]. Fourthly they should be stable, and finally they should be relevantly named to aid effective communication. These same principles were identified by other authors [22] who posited that the classification system itself should be based on key characteristics of the observed phenomena, be more general rather than special purpose, be parsimonious, hierarchical in nature, and timeless. The attributes identified by both McCarthy [20] and Chrisman et al [22] were adopted for the current study, with the exception of Chrisman et al’s [22] hierarchical nature. Whilst hierarchy might be appropriate in objective or systems studies, it is not appropriate for the present study given the subjective nature of the experience of harm.

### Financial harms

The first classification is that of financial harms to the person who gambles, the affected other or the community. At a community level, these may also be referred to as economic harms. Financial harms were a dominant theme throughout all the data, they were normally the first harm mentioned by participants, and a theory of why this occurred was generated that identified three factors. Firstly, financial harms are the trigger for a temporal point of significance, normally a change in a behaviour, reassessing the view of a person or relationship, or seeking assistance and or treatment. Secondly, financial harms are easily identified. The data contained many examples of reported estimates of total financial loss, overall spending patterns, and individual occasion losses. Thirdly, financial harms often had an immediate impact, a significant impact, or were the first order harm that triggered further harms across other dimensions. Given these factors it was unsurprising that financial harms were such a dominant theme.

There was a clear identification of different levels of severity in terms of financial harm within the general harms category. The first level could be described as the loss of surplus; those items or activities that are purchased beyond necessities with surplus or discretionary income or financial resources. These harms related to the loss of capacity to purchase luxury items such as holidays or electronic equipment. This could be seen as a standard purchasing decision; a choice by a rational person to prioritise the purchase of gambling products over other items from discretionary income. However, instances were identified where this had changed from a deliberate informed choice to a process of automaticity by the person who gambles. The choice was often followed by regret and the impact of the choice may also harm affected others.

Also identified in this first level of severity was the erosion of savings and financial resources and the capacity to spend on other discretionary, but not luxury, items such as family outings or social activities, involvement in artistic, cultural, sporting or educational activities. Similarly, the losses had an impact on affected others who were not involved in the choice, and who identified it as an instance of harm. Within this group of harms, it was the loss of rational choice, and the influence of automaticity or sense of loss of awareness or control that made these harmful to both the person who gambles or affected others.

The second group of general financial harms related to activities undertaken to manage short term cash flow issues by either the person who gambles or an affected other. These harms impacted on those who had limited or no surplus income or financial resources prior to engaging with gambling, or those who were consuming gambling products to the level of exhausting their surplus income or financial resources. The activities within this group could be divided into two strategies of managing short term cash flow: funds generation or debt generation.

Examples of the former include undertaking additional employment or selling household items by both people who gamble and affected others. Whilst again these could be argued to be rational financial choices, they were reported as something people were compelled to do and often the source of second and further order harms. This was due to the impact of stress and of time spent at additional employment activities. There were also strong links to second or further order harms in terms of relationship strain, decrements to health, cultural practices and impact on primary employment.

The second strategy for managing short term cash flow was debt generation. Examples include accessing more credit, kite-flying (use of one line of credit to cover the minimum payments on another), pawning items, and non-payment of accounts such as utilities and rates. The liability created by the increase in personal debt and the risk that it creates to financial security was seen as a primary harm. The additional cost of particular credit facilities such as pay-day loans and pawning were highlighted within the data as creating compound financial harms. The use of high risk or illegal credit facilities were also identified. Second and further order harms were linked to debt generation due to the impacts on relationships, stress, physical health, cultural practices and the vulnerability (or risk) it created in terms of emotional distress and involvement in criminal activity.

The third group of general financial harms identified related to the reduction or loss of ability to meet expenditure that had a non-immediate consequence. This included opting out or non-payment of insurance (health, home, income protection, car), non-essential repairs and maintenance of assets such as homes and cars, preventative health activities such as dental check-ups, the purchase of non-essential medication, or utilisation of allied health support. This group represents a harm in terms of either risk or vulnerability, that is, it may not have an immediate impact but creates the risk or vulnerability to a significant later impact, or it created a more incremental lagged impact. For example, whilst the loss of insurance may not have an immediate negative effect, if it is needed it can have a significant detrimental impact that creates risk. The value of assets such as homes and cars are eroded by not maintaining or repairing them, and it can also create a risk of more significant harm where this contributes to an increased risk of injury (i.e., faulty electrical wiring in a home, bald tyres on a car). Similarly the loss of health promotion or screening activities creates risk and vulnerability for more significant and costly impacts later; for example a neglected filling becomes a tooth extraction, or an injury not managed with allied health support creates long term disability.

The final group of general financial harms were related to the reduction or loss of ability to meet expenditure that had immediate consequence. These included the inability to purchase food, essential medications, clothing, health care services, housing, children’s education requirements, and transport costs. It also included the loss of utilities such as heating or water where previous attempts to manage credit options had failed. These harms in addition to having immediate consequences, often created second and further order harms. These included causal sequences such as impacted ability to engage in education or work due to lack of food, inability to attend education or work due to lack of transport, decrements to health due to inappropriate clothing for the climate, or damage to children’s feet due to poorly fitting shoes. For affected others there was a strong causal link to emotional and psychological distress due to the feelings of being unsafe or the inability to control the situation.

The financial group of harms highlighted the subjectivity of the nature of harm due to some experiences being able to be tolerated or accommodated by some, but creating a crisis threshold for others. Treatment and assistance providers consistently identified the financial harms as the tipping point for seeking assistance. However the point at which each individual or family could no longer tolerate a harm (or harms) and would seek assistance varied and may be influenced by how normalised deprivation or poverty already was to them. This threshold was also mediated by informal support networks from families and communities.

As well as the threshold for seeking assistance being related to an inability to tolerate a magnitude of deprivation (such as food, heating, housing, transport), the loss of a significant asset (home, business), the inability to access funds, or bankruptcy, the threshold or crisis could also represent the combination with the impact of a second order harm such as relationship breakdown, extreme emotional distress, suicidal ideation or criminal activity. The threshold or crisis harms were linked to a change in behaviour, albeit only temporary or assistance seeking behaviour. In some cases the change for the person who gambles would include an ongoing effort to reduce, control or abstain from gambling behaviours. However the patterns of changes to gambling behaviours and subsequent harms were variable, which is consistent with earlier empirical studies [18, 19].

Financial harms had a profound impact from a legacy perspective, even when the person who gambled ceased to engage in the activity. Harms identified in the data included the long term impacts of poor credit ratings, financial vulnerability and poverty. Poor credit ratings often attracted higher costs of security bonds, and a reliance on more expensive credit products or pay as you go options which created a compound and ongoing financial harm. When the overall financial harm was of a large magnitude or experienced by an already financially vulnerable individual or family, the impact was strong enough to create a second order harm labelled as a lifecourse or intergenerational harm, such as tipping them into the poverty cycle or homelessness. Second order consequences from a legacy perspective of financial harm included people remaining in relationships they would otherwise leave due to the inability to establish themselves as viable separate households. This was described by one participant as being bound by debt.

### Relationship disruption, conflict or breakdown

The second dimension of harms that emerged from the data was those relating to the relationships between people who gamble and their affected others (including family, friends and community). Similar to financial harms, another key threshold in seeking assistance or treatment was identified where harm to a primary relationship had caused a breakdown of that relationship or a threat to end that relationship. Whilst not able to be quantified as easily as financial harms, this crisis point reflected the importance of the harms to relationships to both the person who gambled and affected others. Relationship harms were often a second order harm due to the consequences of financial harms, but also a primary harm due to the loss of available time of the person who gambles, differences in levels of engagement (attachment/detachment) in the relationship, breaches in trust, and distortion of relationship roles. The experience of the harms was characterised by disruption, where the normal or healthy functioning of a relationship was impacted; conflict, where the impact on the relationship manifests in expressed forms of disagreement or argument; and breakdown, where the relationship has ended or the parties are estranged.

Relationship harms were often strongly linked to the loss of time spent by a person gambling. These included the reduction of the amount of time available or spent with a partner, spouse, child, family member or friend due to engagement with gambling where the loss of that time spent has a negative impact on one or more parties. From this perspective the harms are not unique to gambling and could be seen as similar to any other recreational activity. The loss of time was identified as ranging from episodic to pervasive with the sense of harm also varying based on individual characteristics of both the person who gambles and the affected other. Where the loss of time spent with an affected other would manifest into a second order harm of neglect of a minor or person in their care, this was captured under the dimension of criminal activity as an act of negligence.

Relationship harm caused by the loss of trust within the relationship were strong sub-themes within this dimension. The loss of trust is difficult to objectively determine or measure, yet featured prominently in the data around relationships. It was the source of conflict and breakdown of relationships, and particularly pervasive within the legacy category of harms. Similar to trust, and equally difficult to determine or measure, was the identification of inequality in the amount of engagement or effort put into a relationship. This was particularly evident in the data from affected others both in interview and from forum posts. Whilst the person who gambles might be present and spending time with them they would be reported as being distracted or withdrawn. Similarly the experience of the affected other being more withdrawn and less engaged as a form of punishment of the person who gambles was also reported.

Beyond issues of time and trust, harm to the relationship also stemmed from the personal or cultural perceptions of gambling as a deviant or unacceptable behaviour. In these instances even infrequent recreational engagements with gambling products could create disruption or conflict within a relationship. Second order harms relating to shame and stigma were closely related with these instances of relationship harms.

Where gambling was at diagnostically problematic levels a separate category of relationship harm was identified in terms of relationship distortion. This included the child(ren) of a person who gambles assuming a parent role, with treatment professionals reporting instances of adult children taking on carer roles in terms of financial management tasks and the provision of food or other necessities. They reported instances of minor children having to take care of household tasks and younger children and children staying home from school to try to stop parents from engaging with gambling. This is consistent with impacts reported from other addictive behaviours. Adult children who were experiencing harm from their gambling and returned to their parents’ homes (with our without partners and children) also identified a distortion of the relationship in a form of infanticization. Spouses and partners of people who gambled at problematic levels also reported their relationship having changed to that of a parent/child nature, with them having to assume responsibility for all finances, checking on whereabouts and issuing allowances. A number of informants expressed second order harms of resentment or distress at having to adopt these behaviours with one informant likening it to a form of economic domestic violence due to the levels of control they had needed to assume.

From a legacy perspective relationship harms were reported as very impactful for both the person who gambled and the affected others. Whilst financial losses were of significant initial impact, they could be normalised or adapted to more than relationship losses. Relationship or family breakdowns had significant consequences including social isolation, vulnerability to harmful adaptive behaviours, contribution to emotional or psychological distress, lifecourse and intergenerational harms. Across the data there was a consistency in the focus placed on the ongoing impact.

### Emotional or psychological distress

Harms relating to emotional and psychological distress were also consistently reported, occurring as both primary and secondary or further order harms, and often exacerbated by the impact of other harms. Three sub-themes relating to this classification were identified for both the person who gambles and affected others: emotional and psychological distress from feeling a lack of control over behaviour or circumstance;, feelings of insecurity or lack of safety; and feelings of shame and stigma.

Both the person who gambles and affected others reported distress caused by feelings of lack of control where gambling behaviours had escalated to problematic levels. For the person who gambles this related to the experience of distorted cognitions or erroneous beliefs, feelings of powerlessness in being able to manage gambling behaviours, and desperation in trying to recoup losses. The affected others reported similar experiences of powerlessness relating to their inability to control or influence the behaviour of the person who gambles or the impacts from that gambling, such as financial losses. Control within relationships between people who gamble and affected others were often reported to operate on a type of continuum, with a move to either end often resulting in emotional or psychological harms. Where the affected other felt they did not have control there would be reports of distress or anxiety, but where they were given (or had taken) control within the relationship (normally of finances) this also created harm. At this end of the spectrum the reported emotional harms centred on the experience of resentment or discomfort.

Linked strongly to the theme of control was the sense of security or safety. This is due to the link between feeling in control of one’s future and a sense of safety or security. However, other experiences of emotional or psychological harms around physical safety were identified by participants, relating to harassment by creditors (both legal and illegal). A separate sub-theme related to the idea of being safe from gambling products for those who had experienced problems with their gambling. This was reported as a sense of the invasion of these products into the safety of the home through online product offerings. People who had implemented harm minimisation strategies of self exclusion and actively avoiding land based gambling venues, felt their homes had previously been a safe place where there was not the need for the psychological effort required to resist the urge to gamble. However, the pervasive nature of advertising and links to online gambling through mediums such as social media, coupled with the ineffectiveness of blocking programs or applications as a form of online self exclusion, had removed that feeling of safety and created distress.

Shame and stigma were the most pervasive types of emotional and psychological distress. They existed as initial harms, second or further order harms and affected both the person who gambles and affected others. They could be experienced at any level of participation in gambling, reflecting the link to social and cultural values surrounding gambling. Those experiencing problems with gambling often experienced shame and stigma at more intense levels and these were strongly linked to suicidal ideation and attempts. From a legacy perspective, these harms were particularly impactful especially when linked to other legacy harms such as financial harm and relationship breakdowns, and they created further harms through the manifestation of a lack of self worth, such as decreased levels of self care.

In smaller communities the impact of the stigma for those experiencing problems with gambling was described as a scarlet letter by some participants. The level of stigma directed toward gambling was particularly evident through reports of people who were incarcerated choosing to identify as drug addicts rather than problem gamblers. This shows that even in prison, where antisocial behaviour is normalised to a degree, problems with gambling are still subject to stigma. The notion of shame is also particularly strong in some cultural groups, and was both felt by, and directed at, the whole family. The legacy impact of shame on others was identified as being particularly strong, with some reports of the shame (damage to the family name) as being experienced even by subsequent generations.

### Decrements to health

The harms caused through decrements to biophysical health are not well captured or measured, despite occurring even at recreational levels of gambling. Concerns were expressed by health professionals that gambling represented another sedentary behaviour contributing to the prevalence of that risk factor often in already at-risk populations. Within the data there were links to other risk factors such as smoking, alcohol consumption, and poor nutrition. In more problematic cases gambling was linked to poor sleep practices, non-compliance with medication, and reduced personal hygiene. These behaviours were seen to create both short term impacts, such as headache and migraine relating to focussing on a screen for extended periods of time, but of most concern was their contribution in the long term to increasing risk, creating gateway effects or exacerbating existing comorbidities, particularly chronic disease such as diabetes and depression. These long term impacts also represented legacy harms. This was highlighted as a concern by health professionals particularly for those people who had started or increased their engagement with gambling as a recreational activity due to inability to undertake other recreational activities due to illness, injury or the impacts of aging. Affected others, particularly children, were also impacted often through the lack of available funds and the subsequent impact that has on a number of health determinants.

The biological manifestation of emotional and psychological distress, such as increased blood pressure or loss of sleep, was identified as another form of harm. The impact of this was felt by many participants to be underestimated and rarely captured in current health measures and was experienced by both the people who gamble and affected others. Treatment providers recounted experiences of clients whose deaths had been attributed to causes such as cardiovascular disease, but felt their gambling should have been recorded as a contributing or underlying condition. Similar examples included emergency department presentations for mental health issues, complications due to non-compliance with medication or medical interventions, and injuries caused by violence (including intimate partner violence).

As a consequence of other harms (both individual and cumulative) gambling was identified as contributing to self harm, suicidal ideation, suicide attempts and suicide completions. Levels of these behaviour were anecdotally reported by treatment providers as being higher in people experiencing problems with gambling than those experiencing problems with alcohol and drugs. These types of harm were often linked to treatment seeking and represented a threshold or crisis in terms of harm. They also created ongoing decrements to health as a legacy harm, even if engagement with treatment or assistance had a positive effect.

### Cultural harm

Cultural harms were identified as a separate theme to relationship harms even though they tended to occur together due to the link between family and culture. A person’s culture is more than just the relationship with other people who share the culture, but is grounded in their cultural beliefs, practices and roles. Whilst not strongly represented within the data due to the homogeneity of the participants, there was sufficient reporting of experiences to identify this classification. Harms reported included the dissonance between engaging with gambling where it was against cultural beliefs, the impact of the time spent gambling on the ability to participate in cultural practices and roles, reduction in the ability to contribute or meet the expectations of a cultural community, and the subsequent reduction of connection to the cultural community. Second order harms from this were around experiences of social isolation due to reduced connection, and specific types of shame relating to cultural roles and expectations. Extreme emotional distress was also reported due to a feeling of lost identify due to lost connection with community.

Cultural harms were not isolated to the person who gambles, and were experienced by affected others. This is not unexpected given the important role of family in most cultures. In some cases the harm could be felt by the affected other before the person who gambles. For example, where the affected other was unable to attend events due to the actions of the person who gambles, or their sense of shame at the absence of the person who gambles. Likewise the affected others could also experience social isolation due to lost connection to culture.

### Reduced performance at work or study

The impact of gambling on workplaces is normally reported in terms of criminal activities relating to fraud and embezzlement to address financial demands relating to gambling. Criminal acts of fraud perpetrated against an employer, educational institution or organisation at which someone might be volunteering were captured in a separate classification of harms. The experiences identified from the data within this theme demonstrated a broader and more pervasive catalogue of harms consistent between people who were in paid employment, studying, and undertaking volunteer work. These were grouped within one theme with each activity representing a form of economic contribution.

Harms were identified that included reduced performance due to tiredness or distraction caused by gambling, and there was a clear sense of intensification if there was an escalation in gambling behaviour. This included primary harms such as increased absenteeism due to time spent gambling or second order harm of absenteeism due to lack of transport or ill health as a consequence of gambling. The experience of ill health could be a second or further order harm itself, and thus the impact on work or study could be a compound harm. Similarly the loss of employment and subsequent loss of wage exacerbated financial harms already being experienced.

Work or study related harm that occurred at a threshold level often related to the co-occurrence of criminal activity against the employer. However, instances were reported within the data of people having their employment terminated due to ongoing poor performance. Termination of employment or study opportunities had long term impacts both in terms of gaining future employment (or study), and also contributed to the exacerbation of other harms due to the impact on the ability to generate income creating significant legacy harms.

In addition, work and study harms were experienced by both people who gamble and affected others. For affected others the harm could occur as a second order harm for example, where being tired and distracted at work or study was the result of emotional or psychological distress.

### Criminal activity

Involvement in criminal acts as a consequence of gambling was reported in relation to people who had experienced problems with gambling rather than those engaged in recreational levels of gambling. The involvement in criminal activity was deemed to be a harm, consistent with the functional definition adopted by the present study, in that it creates a decrement to the health or wellbeing of a person including the perpetrator. Involvement in criminal activity had a harmful impact on both the person who gambles and the affected other, and both were also reported within the data as the perpetrators of criminal activity.

Involvement in criminal activity was mostly reported as a second order harm, most commonly to address deficits of funds available to continue engaging in gambling. Interestingly it was reported as being about sourcing funds for gambling rather than for other purchases. The types of criminal activities formed three clear sub-themes: crimes of negligence such as child neglect, crimes of duress such as drug trafficking or prostitution to repay debts, and crimes of opportunity, including acts from petty theft from family members, illicit lending, and fraudulent efforts to attain funds. Fraudulent efforts included embezzlement from employers, welfare fraud, and systematic efforts to obtain funds from family members.

Where criminal activity was detected, this often created a threshold event that led to the detection of problematic gambling, engagement with the justice system, and attempts to address the problems with gambling. These threshold events triggered further harms of relationship conflict or breakdown, job loss, or incarceration. Incarceration or child neglect where children were removed from the person’s care were deemed as lifecourse and intergenerational harms given the profound impact it had on both the perpetrator and their affected others.

From a legacy perspective criminal activity created considerable harms. These included shame and stigma, the impact of a criminal record, and the impact of custodial sentences on both the perpetrator and affected others. At an individual level the affected others extends to any potential victims of the crime, both financially and emotionally and this varied depending on the nature of the crime committed. These were noted as being of consequence not only from an immediate impact but as having a long term second order impact particularly at an emotional or psychological level.

### Lifecourse and intergenerational harms

Whilst the data clearly identified the complex inter-relationship and multiple causal sequences of individual harms and dimensions, there were times when a particular harm or the cumulative impact of harms was so significant that it created a change in the lifecourse of an individual or individuals, generational loss of an individual or the harm passed between generations. Lifecourse and intergenerational effects are a focus within public health due to the level of impact they have as a determinant of health. There were sufficient instances of this within the data, with consistent characteristics and outcomes that they were identified as a separate classification. From a temporal perspective, they usually occurred as a threshold harm but were, as the label of the classification suggests, pervasive legacy harms for both the person who gambles and affected others.

Examples within the data included the experience of generational loss normally relating to financial security or expected stages of financial achievement, such as the inability to secure, or the loss of, a major financial asset such as a house or superannuation. Generational loss was noted in all groups, from young men who had lost their car and job, to middle aged people who had lost homes and businesses, and retirees who had lost homes and savings. The deferment or avoidance of lifecourse milestones such as engagements, marriages and choices surrounding fertility were also reported, with examples of choices to terminate pregnancies or not have children representing another form of generational loss.

Homelessness, incarceration and removal of children (by government agencies) represented a lifecourse and intergenerational harm. The immediate and ongoing impacts of either were significant for both the person who was incarcerated and any children. Part of the impact was related to, and similar in nature to, a general lifecourse and intergenerational harm of a family entering the poverty cycle. Each of these experiences is within themselves an example of a complex interaction of decrements to the health and wellbeing of a family due to issues such as the impact on socio economic status, access to services, experiences of shame and stigma, and further decrements to health.

The loss of primary relationships and subsequent social connection were also reported and represented both a lifecourse and intergenerational harm. In some cases where an adult child had become estranged from their parents it meant their own children had lost the relationship with their grandparents. Whilst family breakdown can be quantified in terms of measuring the incidence of the harm, the impact of it is more difficult to capture. However, its importance is highlighted by the focus placed on the loss of relationships by those who had experienced it. Similar experiences of loss of social connection were also reported in cases where people had to relocate due to the loss of job opportunities, incarceration, or stigma.

### Community level harms

Potential harm from gambling occurs beyond the person who gambles and their affected others and can impact at a community level. This can occur from engagement at recreational levels but more obviously when gambling is problematic. These harms can represent the cumulative impact of harms to individuals within a community, or more direct harms experienced by the community. Consistent with the public health approach and the adaption of a disease model to understand gambling, a clear theme of the contagion of harm from the individual to family and friends, and then community emerged, noting that the person who gambles is the index case and not the source. When gambling became harmful, the harm was absorbed or born by the person who gambles, and at some point spread to the surrounding family and friends. The harm could again spread out to the community. It is important to highlight that these were not necessarily clear thresholds, or identifiable tipping points. The speed and spread of the harm was particularly variable within the data and influenced by a large range of social and environmental factors.

From a perspective of community level financial or economic harms, there is the impact of increased levels of debt and bankruptcy (and the administration of these), the increased reliance on welfare both in terms of welfare payments from government and support services provided by non-government and community organisations, and from a legacy perspective the perpetuation of poverty and welfare reliance. Examples were also identified in the data of business closures related to embezzlement. This had further impact where there were employees who then lost jobs, and the flow on impact on other businesses that supplied or otherwise interacted with that business.

Financial community level harms included: the costs of relationship breakdowns, particularly marriages and the associated costs to the family law courts, the costs of increased welfare support, and the administration of custodial and financial support. Similar costs were identified relating to other relationship breakdowns, such as older parents or adult children who were not able to access care or support from family members.

Relationship harms at a community level include damage done to social cohesion and social capital through isolation or exclusion of individuals or groups. Whilst this type of harm was usually identified in cases of relationship breakdowns between couples or families, some participants identified divisions within communities based on attitudes to gambling that became harmful when issues such as applications for increased gaming licences were being considered. This example of harm was not unique to gambling, and reflects community experiences on many contentious subjects.

Community level emotional or psychological distress and decrements to health can be described as an increased burden of disease due to the exacerbation of onset of illness related to community members’ engagement in gambling. Beyond the cumulative experience of loss to health there is a cost to the community associated through the need to provide health services, medications and treatment costs, and the opportunity cost of the funds used for these that might be addressing other health issues. However, separating out the contribution to these decrements from other comorbidities or contributing behaviours was beyond the scope of the present study.

Cultural harms at the community level fell into two clear sub-themes. The cumulative impact of individual harms led to the lost contribution (role, time or financial) to the cultural community that created a demand on other members or led to a reduced ability to engage in cultural practices by that community. A second and more direct group of harms were around cultural identity, including the use of cultural norms and practices to promote engagement with gambling, and the disconnection of youth when gambling was against cultural or religious beliefs. Cultural identity was also harmed through the exacerbation of cultural stereotypes, creating feelings of hopelessness and powerlessness through the negative narrative surrounding reporting of gambling behaviours by cultural groups. For indigenous cultures there was a sense of exacerbation of existing harms of cultural loss already experienced from colonisation.

Harms relating to performance in work or study were another dimension that had financial impacts at the community level. Absenteeism and job turnover contribute either direct or indirect costs to the economy, as do businesses that close or have a reduced capacity. Similarly, for education, the reduced engagement or withdrawal from post-secondary education had immediate community level impacts and the long term effect of reduced workforce skills which impacts on employability and economic growth. Volunteer (non-paid) work was included within this dimension due to the direct impact volunteer contributions make to the economy and social capital of communities. Examples were identified in the data where the ability or desire to engage in volunteer work had been impacted by individual’s gambling behaviours.

At a community level criminal activity has very clear impacts. The direct impacts include the costs of the criminal activity in terms of the investigation of crimes or neglect, the costs from the judicial system, provision of incarceration, management of probation and parole or costs of removing and case managing children experiencing neglect. Other direct harms include the cumulative effect on any victims of the crime or neglect, and the families or friends of the perpetrator. Indirectly criminal activity and neglect have strong effects on social capital including social cohesion and feelings of safety.

Life course and intergenerational harms also had cumulative impacts at a community level. These again were largely related to economic impacts or loss of social capital. A strong theme within the data was that the normalisation of gambling and the pervasive embedding of gambling other activities such as sport, was a community level intergenerational harm.

### Taxonomy of harms

A taxonomy of the specific harms that were identified within the data was created; aiming to facilitate the development of more robust measurements of gambling harm, for use in developing policy in relation to harm minimisation and prevention, and as a potential tool for treatment and support professionals in assisting clients to unpack individual experiences and identify complimentary support services. This was separated into three separate taxonomies of gambling harm that are included as Tables 1, 2 and 3. The separation reflects the differentiation of harms experienced by the person who gambles, affected others and the broader community. The taxonomies for the person who gambles and the affected others reflect the proposed conceptual framework whilst the community level harms reflect the classifications but not the categories of the conceptual framework. This is because the community level harms represent a collective or population level experience, not an individual one, making the temporal categories inappropriate.

**Table 1:** A taxonomy of harms experienced by people who gamble

	General	Crisis	Legacy
**Financial Harm**	• Reduction or loss of capacity to purchase luxury items such as holidays, electronics • Reduction or loss of discretionary spending such as non-gambling related entertainment or other family members’ activities (ie. children’s sports) • Erosion of savings • Activities to manage short term cash-flow issues: o Additional employment or other forms of income generation o Accessing more credit o Use of credit cards (kite flying) o Selling or pawning items o Pay day loans o Non-payment or juggling of large bills such as utilities or rates • Cost of replacing items sold or pawned as part of short term cash strategies • Reduction or loss of non-immediate consequence expenditure o Insurance (health, home, car, income protection, business) o Repairs or maintenance costs (home, car, business) o Health promotion activities (check-ups, long term medications, allied health support) o Household items • Reduction or loss of expenditure on items of immediate consequence: o Children’s expenses (education) o Medication or health care o Clothing o Food (including use of food parcel) o Housing or accommodation o Needing assistance with bill payments from welfare organisations or inability to pay bills (eg utilities) o Transport costs (petrol, fares)	• Loss of sources of additional funds (ie no further credit available) • Loss of capacity to meet requirements of essential needs (food) • Loss of normal accommodation requiring temporary accommodation or resulting in homelessness • Loss of major assets (car, home, business) • Bankruptcy	• Reliant on welfare • Restrictions due to bankruptcy or credit rating • Ongoing financial hardship • “Forced” cohabitation or involvement in unhealthy relationship due to financial constraint • Further financial harm from attempts to manage debt (ie. Non-reputable finance providers for debt consolidation) • Ongoing issues relating to financial security, poverty, or financial disadvantage. • Higher costs associated with poor credit rating including premium cost of pay as you go services or increased security bonds.
**Relationship Disruption, Conflict or Breakdown**	• Dishonest communication within relationships with spouse, partner, children, family, friends or community • Unreliable or unavailable to spouse, partner, children, family, friends or community • Reduced amount of time spent with spouse, partner, children, family, friends or community • Reduced quality of time spent with spouse, partner, children, family, friends or community • Disengagement or withdrawal from relationship responsibilities. • Increased levels of neglect of relationships • Pervasive neglect or disengagement from relationships • Reduced engagement in family or social events, • Tension with spouse, partner, children, family, friends or community • Minor or occasional conflict due to increased involvement in gambling or suspicion of increased involvement with gambling • Serious or regular conflict due to increased involvement in gambling or suspicion of increased involvement with gambling • Major or constant conflict due to increased involvement in gambling or suspicion of increased involvement with gambling • Loss of trust from relationship with spouse, partner, children, family, friends or community • “Punishment” by spouse, partner, children, family, friends or community • Episodic distortion of relationship roles (infantilising the person gambling, others including children having to take parental type role) • Incidence or escalation of family violence or intimate partner violence	• Threat of separation or rejection from relationship with spouse, partner, children, family, friends or community • Actual separation or rejection from relationship with spouse, partner, children, family, friends or community • Social isolation • Loss of relationship (temporary or permanent) with spouse, partner, children, family, friends or community • Distortion of relationship roles (infantilising the person gambling, others including children having to take parental type role) • Incidence or escalation of family violence or intimate partner violence	• Social isolation due to ongoing estrangement from relationships with spouse, partner, children, family, friends or community • Vulnerability to problematic gambling relapse due to isolation or relationship breakdown • Inability or reluctance to participate in social functions at gambling venues • Ongoing “punishment” or resentment from spouse, partner, children, family, friends or community • Relationship rebuilding or reconciliation • Ongoing involvement of family court in parenting or co-parenting • Long term damage or estrangement from relationship/s with spouse, partner, children, family, friends or community • Ongoing distortion of relationship roles (infantilising the person gambling, others including children having to take parental type role) • Loss of psychological development through lack of appropriate social interaction • Incidence or escalation of family violence or intimate partner violence
**Emotional or Psychological Distress**	• Emotional and psychological distress caused by living outside of your value system • Experience of distorted cognitions or erroneous beliefs • Emotional or psychological distress of hiding gambling from others (including lying and creating alibis for lost time and money) • Reduced feelings of self-worth and pride • Increased feelings of shame • Increased feelings of inadequacy or personal failing because of inability to control gambling to recreational levels • Perceptions of being stigmatised • Emotional or psychological distress of inability to control gambling • Increased feelings of insecurity and vulnerability • Emotional or psychological distress caused by other harms • Emotional or psychological distress due to harm caused to others (guilt) • Loss of “face” or reputation due to impact of other harms • Desperation from not being able to recoup losses. • Emotional or psychological distress of not wanting to accept problems with gambling • Loss of sense of future or ability to get ahead • Increasing feelings of powerlessness • Fear and distress from follow up and harassment by creditors (legal and illegal)	• Extreme emotional or psychological distress in relation to other harms • Extreme emotional or psychological distress due to harm caused to others • Extreme emotional or psychological distress caused by living outside of your value system • Complete loss of feelings of self-worth and pride • Extreme shame • Extreme sense of hopelessness and powerlessness • Suicidal ideation • Loss of “face” or reputation (stigma) if problem with gambling becomes publicly known • Shame from utilising responsible gambling measures such as exclusion or seeking treatment. • Extreme fear and distress from follow up and harassment by creditors (legal and illegal)	• Experienced, perceived and internal stigma • Ongoing guilt and shame • Emotional and psychological impacts of managing recovery or harm minimisation strategies including constant vigilance and behavioural adaptation • Ongoing feelings of insecurity and vulnerability • Ongoing emotional and psychological distress in relation to other harms • Ongoing emotional or psychological distress due to harm caused to others • Ongoing emotional or psychological distress caused by having lived outside of your value system • Ongoing vulnerability to suicidal behaviours
**Decrements to Health**	• Increased sedentary behaviour during time spent gambling • Biological manifestation of emotional and psychological distress eg. increased blood pressure, loss of sleep • Reduced levels of self-care: o nutrition o hygiene o sufficient sleep o compliance with medical care o physical activity o reduced quality of living circumstances (ie. cannot afford heating) • Incidence of disease or injury due to reduced levels of self care • Increased risk due to gateway effect, interaction with, or exacerbation of other health risk factors (drinking, smoking, illegal substances) • Increased risk due to gateway to, interaction with, or exacerbation of comorbidities (depression, anxiety, biophysical chronic disease) • Increased experience of family violence due to involvement in gambling • Incidence of self-harm • Minor health ailments (headache migraine) relating to focussing on a screen for long periods of time with particular gambling products	• Physical impacts of living rough due to homelessness, including increased risk of disease, violence and impact of poor living conditions • Experience of violence due to involvement in gambling • Medical emergency (including mortality) due to onset, exacerbation, or failure to diagnose condition due to gambling • Serious self-harm • Attempted (or completed) suicide	• Ongoing disability or decrement to health through attempted suicide or other forms of self-harm • Ongoing increased risk of disease or decrement to health due to legacy effects of risk factors or poor self-care • Ongoing disability or decrement to health due to other medical conditions exacerbated or advanced due to involvement with gambling.
**Cultural Harm**	• Reduced engagement in cultural rituals • Culturally based shame in relation to cultural roles and expectations • Reduction of contribution to community and cultural practices of the community • Reduction of cultural practices • Reduced connection to cultural community • Harm to individual through reduced connection to community and culture in terms of increased social exclusion or isolation	• Extreme cultural shame in relation to culturally based roles and expectations • Loss of ability to contribute to community • Impact (loss) on cultural practices • Damaged or lost connection to community and culture • Harm to individual through reduced or lost connection to community	• Ongoing cultural shame in relation to roles and expectations • Ongoing reduction or loss of contribution to community • Ongoing reduction or loss of cultural practices • Ongoing loss of connection to community • Ongoing harm to individual through reduced connection to community
**Reduced Performance at Work or Study**	• Reduced performance due to tiredness or distraction • Increased absenteeism due to time spent actually gambling, tiredness, ill health or lack of transport due to gambling • Workplace or educational institution consequences of use of work or educational institution resources for gambling activity • Reduced availability to contribute to the community through volunteer work	• Loss of job due to theft or fraud involving employment or educational institution • Loss of job, suspension or exclusion from educational institution due to poor performance • Exacerbation or contribution to other harms due to job loss (including loss of wage) • Rejection from volunteer work	• Reduced opportunity for employment or enrolment due to past poor performance or criminal activity • Ongoing impact in participation in volunteer work (linked to reputation and restriction of activities)
**Criminal Activity**	• Vulnerability to illegal activities that can provide fast access to funds • Engagement in crimes of negligence - acts such as child neglect (leaving children unsupervised) • Engagement in crimes of opportunity - petty theft including from family members • Engagement in crimes of opportunity - property crimes for funds, illicit lending, fraudulent efforts to attain funds • Engagement in crimes of duress - relating to repaying debt such as drug trafficking and prostitution	• Arrest and/or conviction of criminal activity of opportunity • Arrest and/or conviction of criminal activity of duress • Arrest and / or conviction of criminal activity of negligence	• Impact of criminal record on future employment opportunities, voluntary and community opportunities, travel restrictions • Disruption to relationships of custodial sentence • Ongoing impact on spouse, partner, child, family and friends due to impact of criminal record or custodial sentence through other mechanisms • Trans-generational impact of criminal record or custodial sentence • Shame and stigma of criminal conviction or involvement in criminal activity
**Lifecourse and Intergenerational Harms** • Generational loss relating to financial security or stages of financial achievement (ongoing impact caused by inability to secure or loss of major asset, superannuation) • Loss of lifecourse events such as engagement/marriage/having children (generational loss) • Loss of primary relationships and social connection (including parents/children/community) • Having to move towns/states due to impact of gambling or other harms • Homelessness • Change to career due to impact of gambling or other harms • Incarceration due to gambling

**Table 2:** A taxonomy of harms experienced by affected others of people who gamble

	General	Crisis	Legacy
**Financial Harm**	• Additional costs due to lack of capacity of person who gambles to meet their costs or joint costs (minor to major items) • Reduction or loss of capacity to purchase luxury items such as holidays, electronics • Reduction or loss of discretionary spending such as non-gambling related entertainment or other family members’ activities (ie. children’s sports) • Erosion of savings • Activities to manage short term cash-flow issues: o Additional employment or other forms of income generation o Accessing more credit o Use of credit cards (kite flying) o Selling or pawning items o Pay day loans o Non-payment or juggling of large bills such as utilities or rates • Cost of replacing items sold or pawned as part of short term cash strategies • Reduction or loss of non-immediate consequence expenditure o Insurance (health, home, car, income protection, business) o Repairs or maintenance costs (home, car, business) o Health promotion activities (check-ups, long term medications, allied health support) o Household items • Reduction or loss of expenditure on items of immediate consequence: o Children’s expenses (education) o Medication or health care o Clothing o Food (including use of food parcel) o Housing or accommodation o Needing assistance with bill payments from welfare organisations or inability to pay bills (eg utilities) o Transport costs (petrol, fares)	• Loss of capacity to meet requirements of essential needs (food) • Loss of normal accommodation requiring temporary accommodation or resulting in homelessness • Loss of major assets (car, home, business) • Bankruptcy	• Reliant on welfare • Restrictions due to bankruptcy or credit rating • Ongoing financial hardship • “Forced” cohabitation or involvement in unhealthy relationship due to financial constraint • Further financial harm from attempts to manage debt (ie. Non-reputable finance providers for debt consolidation) • Ongoing issues relating to financial security, poverty, or financial disadvantage. • Higher costs associated with poor credit rating including premium cost of pay as you go services or increased security bonds.
**Relationship Disruption, Conflict or Breakdown**	• Dishonest communication within relationship from person who gambles to affected other • Person who gambles is unreliable or unavailable to affected other • Reduced amount of time spent with person who gambles • Reduced quality of time spent with person who gambles • Feelings of unequal contribution to relationship with person who gambles • Disengagement or withdrawal from relationship responsibilities by person who gambles • Increased levels of neglect of relationship by person who gambles • Reduced engagement in family or social events with person who gambles, • Tension in relationship with person who gambles • Tension in other relationships due to emotional and/or material demands of trying to manage relationship with person who gambles • Minor or occasional conflict due to increased involvement in gambling or suspicion of increased involvement with gambling by person who gambles • Serious or regular conflict due to increased involvement in gambling or suspicion of increased involvement with gambling by person who gambles • Major or constant conflict due to increased involvement in gambling or suspicion of increased involvement with gambling by person who gambles • Loss of trust from relationship with person who gambles • Episodic distortion of relationship roles (infantilising the person gambling, others including children having to take parental type role) • Significant disruption to other relationships due to emotional and/or material demands of trying to manage relationship with person who gambles • Episodic distortion of relationship between affected others (ie. Spouse of person who gambles using children of relationship as confidant) • Incidence or escalation of family violence or intimate partner violence	• Contemplation of separation or rejection from relationship with person who gambles • Actual separation or rejection from relationship with person who gambles and potentially related others • Loss of other relationships due to emotional and/or material demands of trying to manage or remaining in relationship with person who gambles • Social isolation due to feelings of shame or being stigmatised • Loss of relationship (temporary or permanent) with spouse, partner, children, family, friends or community • Distortion of relationship roles (infantilising the person gambling, others including children having to take parental type role) • Incidence or escalation of family violence or intimate partner violence	• Feelings of guilt over ending relationship with person who gambles and potential impact • Social isolation due to ongoing estrangement from other relationships • Vulnerability to continuing in ongoing unhealthy relationship with person who gambles (episodic reconciliations) for reasons of guilt or inadequacy • Inability or reluctance to participate in social functions at gambling venues to protect person who gambles • Ongoing resentment and shame within relationship with person who gambles • Relationship rebuilding or reconciliation • Ongoing involvement of family court in parenting or co-parenting • Long term damage or estrangement from person who gambles and potentially related others • Ongoing distortion of relationship roles (infantilising the person gambling, others including children having to take parental type role or confidant role) • Inability to form trusting relationships with others or hypervigilance within relationships • Incidence or escalation of family violence or intimate partner violence
**Emotional or Psychological Distress**	• Feelings of frustration over person who gamble’s behaviour • Anxiety when person who gambles does not respond to normal communication methods • Emotional and psychological distress caused by difference to own value system • Emotional or psychological distress of feelings of suspicion or being lied to • Reduced feelings of self-worth • Feelings of shame or guilt • Loss of feeling safe and secure in life • Increased feelings of inadequacy or personal failing because of inability to help person who gambles • Emotional or psychological distress from being manipulated or threatened (threats to the affected other or threats of self harm by person who gambles) • Perceptions of being stigmatised • Anxiety when person who gambles disappears for extended periods of time without contact (days) • Emotional or psychological distress of being blamed for other person’s gambling • Emotional or psychological distress at people arguing because of gambling behaviours (children) • Increased feelings of insecurity and vulnerability • Emotional or psychological distress caused by other harms • Loss of “face” or reputation due to impact of other harms • Loss of sense of future or ability to get ahead • Increasing feelings of powerlessness • Guilt over harms to other affected others • Increased feelings of anger and frustration • Fear and distress from follow up and harassment by creditors (legal and illegal) • Feelings of guilt if affected other was the person who introduced the person who gambles to gambling • Increased risk to emotional or psychological wellbeing of affected other in the care of the person who gambles due to their distraction or tiredness	• Extreme emotional or psychological distress in relation to other harms • Extreme emotional or psychological distress due to harm caused to other affected others • Extreme emotional or psychological distress caused by living in constant feelings of insecurity and vulnerability • Complete loss of feelings of self-worth and pride • Extreme shame • Extreme sense of hopelessness and powerlessness • Emotional or psychological distress of dealing with person who gambles problems including their distress, self harm, suicidal ideation or completion. • Loss of “face” or reputation (stigma) if person who gambles’ problem with gambling becomes publicly known • Emotional or psychological distress of supporting and/or assisting person who gambles to seek treatment • Extreme fear and distress from follow up and harassment by creditors (legal and illegal) • Grief and/or resentment for loss of security, lifestyle, relationship • Feelings of rejection that gambling is chosen over them	• Experienced and perceived stigma • Ongoing guilt and shame • Emotional and psychological impacts of supporting recovery or harm minimisation strategies including constant vigilance and behavioural adaptation • Ongoing feelings of insecurity and vulnerability • Ongoing emotional and psychological distress in relation to other harms • Ongoing emotional or psychological distress due to harm caused to other affected others • Ongoing emotional or psychological distress of vigilance to mental health status of person who gambles including distress, self harm, suicidal ideation or completion • Ongoing feelings of grief, resentment and anger
**Decrements to Health**	• Physical impacts of other harms • Biological manifestation of emotional and psychological distress eg. Feeling tired, increased blood pressure, loss of sleep, migraine, nausea, diarrhoea • Reduced levels of self-care: o nutrition o hygiene o sufficient sleep o compliance with medical care o physical activity o reduced quality of living circumstances (ie cannot afford heating) • Incidence of disease or injury due to reduced levels of self care • Increased risk due to gateway effect, interaction with, or exacerbation of other health risk factors (drinking, smoking, illegal substances) • Increased risk due to gateway to, interaction with, or exacerbation of morbidities (depression, anxiety, biophysical chronic disease) • Increased experience of family violence due to involvement with person who gambles • Incidence of self-harm • Increased risk to physical wellbeing of affected other in the care of the person who gambles due to their distraction or tiredness	• Onset of health condition due to exacerbation of risk factors or continued stress from other harms • Physical impacts of living rough due to homelessness, including increased risk of disease, violence and impact of poor living conditions • Experience of violence due to involvement with person who gambles • Medical emergency (including mortality) due to onset, exacerbation, or failure to diagnose condition due to impacts of person who gamble’s behaviours • Serious self-harm • Attempted (or completed) suicide	• Ongoing disability or decrement to health through attempted suicide or other forms of self-harm • Ongoing increased risk of disease or decrement to health due to legacy effects of risk factors or poor self-care • Ongoing disability or decrement to health due to other medical conditions exacerbated or advanced due to involvement with person who gambles
**Cultural Harm**	• Reduced engagement in cultural rituals • Culturally based shame in relation to cultural roles and expectations • Reduction of contribution to community and cultural practices of the community • Reduction of cultural practices • Reduced connection to cultural community • Harm to individual through reduced connection to community and culture in terms of increased social exclusion or isolation	• Extreme cultural shame in relation to culturally based roles and expectations • Loss of contribution to community • Impact (loss) on cultural practices • Damaged or lost connection to community and culture • Damage to individual through reduced or lost connection to community	• Ongoing (including intergenerational) cultural shame in relation to culturally based roles and expectations • Ongoing reduction or loss of contribution to community • Ongoing reduction or loss of cultural practices • Ongoing loss of connection to community • Ongoing (intergenerational)damage to individual through reduced connection to community
**Reduced Performance at Work or Study**	• Reduced performance due to tiredness or distraction • Increased absenteeism due to time spent supporting or addressing problems of person who gambles • Reduced availability to contribute to the community through volunteer work	• Theft or fraud involving employment or educational institution • Loss of job, suspension or exclusion from educational institution • Exacerbation or contribution to other harms due to job loss (including loss of wage) • Impact on others of loss of job or education	• Reduced opportunity for employment or enrolment due to past poor performance or criminal activity • Trans-generational impact of loss of income and reduced future ability to participate in employment • Ongoing impact in participation in volunteer work (linked to reputation and restriction of activities)
**Criminal Activity**	• Victim of crime from person who gambles – petty theft of items or small amounts of cash. • Vulnerability to illegal activities that can provide fast access to funds • Engagement in crimes of opportunity - petty theft including from family members • Engagement in crimes of opportunity - property crimes for funds, illicit lending, fraudulent efforts to attain funds • Engagement in crimes of duress - relating to repaying debt such as drug trafficking and prostitution	• Victim of crime from person who gambles –fraud • Victim of crime from person who gambles – significant theft of money or items • Victim of crime from involvement of person who gambles in illegal activities • Arrest and/or conviction of criminal activity of opportunity • Arrest and/or conviction of criminal activity of duress • Arrest and/or conviction of criminal activity of negligence	• Ongoing impacts from being victim of crime • Impact of criminal record on future employment opportunities, voluntary and community opportunities, travel restrictions • Disruption to relationships of custodial sentence • Ongoing impact on spouse, partner, child, family and friends due to impact of criminal record or custodial sentence through other mechanisms • Trans-generational impact of criminal record or custodial sentence • Shame and stigma of criminal conviction or involvement in criminal activity
**Lifecourse and Intergenerational Harms** • Delay in life course events and matters of financial security and achievement • Generational loss relating to financial security or financial achievement (ongoing impact caused by loss of major asset, superannuation) • Loss of lifecourse events such as engagement/marriage/having children (generational loss) • Loss of primary relationships and social connection (including parents/children/community) • Homelessness • Having to move towns / states due to impact of person who gambles or other harms • Incarceration

**Table 3:** A taxonomy of harms experienced by communities

**Financial Harm**	**Relationship Disruption, Conflict or Breakdown**	**Emotional or Psychological Distress**	**Decrements to Health**
• Increased reliance on welfare both community and government provided. • Increased levels of debt and bankruptcy (administration of these) • Broader impact to the community of business closures. • Perpetuation of poverty and welfare reliance from a generational perspective. • Redistribution of community funds through biased processes. • Impact on fundraising ventures for community organisations.	• Costs to the family law courts, and associated organisations. • Costs of caring for dependents no longer supported • Damage to social cohesion and social capital through isolation and exclusion.	• Decline in social and cultural capital. • Costs associated with provision of services to assist people with emotional and psychological harms • Burden of disease from related psychological harms • Harms to venue workers.	• Increased costs to the health system (direct and indirect) both in terms of treatment for gambling and costs associated with other medical conditions caused or exacerbated by gambling.
**Cultural Harm**	**Reduced Performance at Work or Study**	**Criminal Activity**	**Lifecourse or Intergenerational Harms**
• Community must make up for lost contributions (roles, time, finance) due to disconnection of members • Use of cultural norms and practices to promote gambling (disrespectful to the culture) • Exacerbation of hopelessness through negative narrative associating culture with gambling problems • Disconnection of youth (generational loss)	• Cost of job turnover, absenteeism. • Impact on employment at other businesses affected by gambling harm (ie. where a business closes and businesses that interacted with it lose sales). • Decreased participation in volunteering and other community activities.	• Direct costs of criminal activity in terms of the investigation of crime, costs to the judicial system, incarceration, probation and parole. • Cost to victims of crime both financial and emotional.	• Normalisation of gambling and gambling related harm • Cumulative impact of generational losses • Transgenerational loss creating dependency

In each of the taxonomies the items are mutually exclusive between classifications, but not categories. The categories assigned within the taxonomies represent the temporal sequence where they were identified within the data, however this data is not representative and cannot be generalised. The subjective nature of a threshold makes generalisation inappropriate and as such it is seen more of a reflection of experiences identified with a harm within the data but would be appropriate to test empirically within a population survey.

The items listed within each of the taxonomies represent broad rather than specific harms to facilitate the operationalisation of measures of harm in future studies. On completion of the taxonomies each identified harm within the data was checked against the items to ensure the individual experience was captured in the generalised items. For example “lied to my mates” is captured by “Dishonest communication within relationships with spouse, partner, children, family, friends or community”.

### Future research

The findings of the current study support the criticisms of previously used proxy measures of gambling related harm as being inappropriate. This is particularly true of gambling behaviour measures such as the PGSI or monetary loss. Whilst these measures have an important contribution to our understanding and examination of gambling as a behaviour, as a health behaviour they should be considered as a risk factor and not as an outcome. A broader understanding and conceptualisation of harm that moves from the current pathogenic approach of a behavioural classification (PGSI) or a diagnostic case (DSM) is consistent with social models of health and necessary if we are to develop ham minimisation strategies that address the full breadth of gambling’s impact. It captures the impact of harm on other determinants of health, both proximal and distal, that have profound impacts on individual and population health over the lifecourse. The findings of this study provide a foundation for developing more appropriate population measures of gambling harm than the current proxy measures offer.

Further research is needed to determine the prevalence of harms within the population who are exposed to gambling, either through their own or someone else’s gambling behaviour. The findings from this study could also be used in the development of summary measures, such as health related quality of life weightings, of the overall impact of gambling on population health allowing the comparison of gambling related harm to other health issues. Longitudinal research is also needed to determine incidence patterns and risk factors associated with the different harms.

## Conclusion

It is important to caveat that the harms outlined in this study can occur due to engagement in other behaviours and can be exacerbated by the influence of comorbidities or existing dysfunction. However, this initial work is aimed to facilitate the understanding of gambling related harm from a much broader perspective than is currently implied by the use of inadequate proxy measures and one that is consistent with moving towards a public health approach to gambling. The WHO definition of health was adopted to ensure that definition, conceptual framework and taxonomy of harms captured the full breadth and impact of gambling. Consistent with an understanding of the determinants of health, gambling as a behaviour can be seen to have an impact on a number of other determinants both proximal and distal that increase risk of, or contribute to, negative health outcomes. The quantification of this influence is beyond the scope of the current study but an important area of future research. The relationship and interaction between these harms and determinants of health are complex and interwoven, and vary significantly between individuals, families and communities.

The contribution of this study has been to identify and organise the diverse impacts on health and wellbeing that can occur as a result of gambling. The seven domains identified provide an organising structure for future research to investigate harms. Whilst it does not follow that each domain necessarily contributes equally to the ‘burden of harm’; each domain should at the least be investigated to ascertain its relative contribution to the experience of harm. A priority for future research on gambling harms is clearly the development of an effective measurement instrument, and the specific harms, and domains identified should assist in this process. The present paper has placed equal weight on the harms suffered by gamblers themselves, and the individuals and community surrounding them. We suggest that any population-based measures of gambling harm should also give these harms to others appropriate attention.
